# Molecular characterisation of infectious pancreatic necrosis viruses isolated from farmed fish in Finland

**DOI:** 10.1007/s00705-017-3525-8

**Published:** 2017-08-09

**Authors:** Riikka Holopainen, Anna Maria Eriksson-Kallio, Tuija Gadd

**Affiliations:** 1 Finnish Food Safety Authority Evira, Research and Laboratory Services Department, Virology Research Unit, Mustialankatu 3, 00790 Helsinki, Finland; 2 Finnish Food Safety Authority Evira, Research and Laboratory Services Department, Veterinary Bacteriology and Pathology Research Unit, Mustialankatu 3, 00790 Helsinki, Finland

## Abstract

Infectious pancreatic necrosis virus (IPNV) has been isolated annually since 1987 from salmonids without clinical signs at coastal fish farms in Finland. In the inland area, viral isolations were rare until 2012, when IPNV was detected at several freshwater fish farms. Between 2013 and 2015, the infection spread and IPNV was continuously isolated from several farms, both inland and on the coast. The aim of this study was to genetically characterise the IPNV isolates collected from Finnish coastal and inland fish farms over the last 15 years, and to detect genetic changes that may have occurred in the virus populations during the study period. The partial VP2 gene sequence from 88 isolates was analysed. In addition, a complete genomic coding sequence was obtained from 11 isolates. Based on the genetic analyses, Finnish IPNV isolates belong to three genogroups: 2, 5 and 6. The genetic properties of the isolates appear to vary between inland farms producing juveniles and food fish farms in the coastal region: the inland farms harboured genogroup 2 isolates, whereas at coastal farms, all three genogroups were detected. Little genetic variation was observed within the Finnish genogroup 2 and 5 isolates, whereas among the genogroup 6 isolates, two subgroups were detected. All isolates studied demonstrated amino acid patterns in the viral VP2 gene previously associated with avirulence. However, increased mortality was detected at some of the farms, indicating that more research is needed to clarify the relationship between the pathogenicity and genetic properties of IPNV isolates from different genogroups.

## Introduction

The genus *Aquabirnavirus* belongs to the family *Birnaviridae*, and classifies viruses that have been isolated from various freshwater and marine fish species, as well as from bivalve molluscs and crustaceans [1, 2]. Viruses classified within the type species of the genus, *Infectious pancreatic necrosis virus* cause highly contagious disease and mortality in juvenile salmonid fish [3–5]. The pathogenicity of infectious pancreatic necrosis virus (IPNV) varies greatly, depending not only on the viral isolate, but also on the age and physical condition of the host animal [6–8]. IPNV is distributed worldwide, and the virus is transmitted both vertically and horizontally [2].

Aquabirnavirus particles are non-enveloped and single-shelled with a diameter of about 60 nm [9]. They have a double-stranded RNA (dsRNA) genome that consists of two linear segments (A and B) [10]. Segment A contains a large open reading frame (ORF) that encodes a 106-kDA polyprotein (NH_2_-pVP2-VP4-VP3-COOH), and a smaller ORF that overlaps the amino-terminal end of the polyprotein ORF that encodes a 17-kDA nonstructural anti-apoptotic protein VP5 [11–13]. The polyprotein is cleaved co-translationally to generate pVP2, a precursor of the major capsid protein VP2, VP4, a protease, and VP3, a minor capsid protein that forms complexes with the genomic RNA inside the viral capsid. The pVP2 precursor is further processed to generate a mature VP2 protein [2]. VP2 is the most abundant protein in the virion and is responsible for the production of neutralizing antibodies in the host [13]. Additionally, it has been shown in several studies that certain VP2 amino acid residues determine the virulence of IPNV strains, and that some of these amino acid positions are highly variable among different strains [7, 14, 15]. Segment B encodes VP1, an RNA-dependent RNA polymerase (RdRp) that is found free in the viral particle, as well as covalently associated with the viral genome [16]. Aquabirnaviruses have traditionally been classified into nine cross-reactive serotypes, A1–A9, based on their antigenic properties [17]. More recently, viral VP2 gene sequences were shown to correlate with the serological classification and geographical distribution, and genogroups 1–7 were defined based on sequence similarities [18, 19].

In the coastal area of Finland, infectious pancreatic necrosis (IPN) was first reported in 1984 [20] and has occurred annually since 1987 on fish farms without causing severe clinical disease. IPNV isolations were extremely rare in continental Finland until 2012, when the virus was detected at several inland farms [21]. The inland IPNV isolates were demonstrated to belong to genogroup 2 based on partial VP2 sequences showing genetic properties associated with low pathogenicity, and the origin of the isolates appeared to be one or several coastal farms.

The aim of this study was to genetically analyse IPN viruses isolated from Finnish fish farms several years before and a few years after the introduction of IPNV in 2012 to inland areas. To achieve this, the partial VP2 gene was sequenced from all isolates studied and a complete coding sequence of the viral genome was obtained from selected isolates using next generation sequencing. The sequences obtained were used to examine the genetic properties of the isolates, including properties associated with pathogenicity, to determine how the IPNV populations have evolved under Finnish fish farming conditions during the past 15 years.

## Materials and methods

### Viral isolates

Altogether, 88 viral isolates were collected from Finnish coastal and inland fish farms during 1989, 2000–2011 and 2014–2015, and analysed for the partial VP2 gene sequence. In addition to VP2 sequences, 11 isolates from the years 1989, 2001, 2007, 2010 and 2012 were analysed for the complete coding region of the genomic segments A and B.

**Fig. 1:**
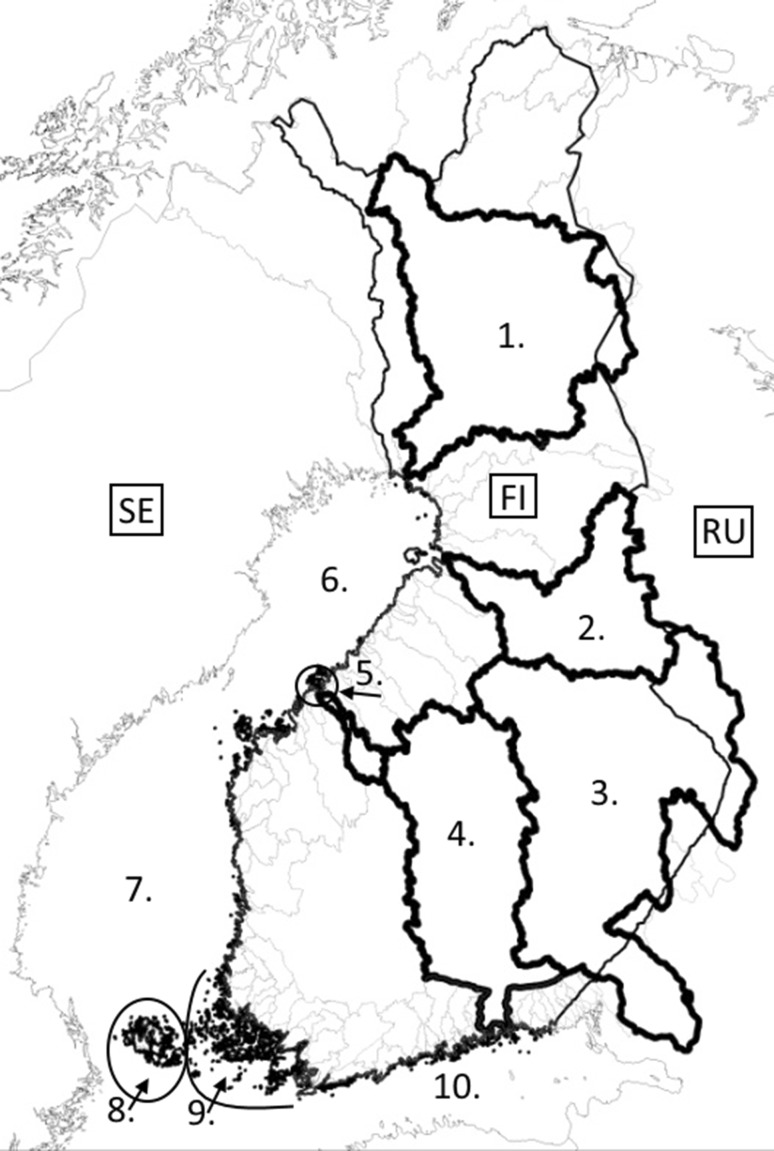
Map showing the geographical areas from which the viral isolates included in the study were obtained. 1 = Kemijoki, 2 = Oulujoki, 3 = Vuoksi, 4 = Kymijoki, 5 = Ähtävänjoki, 6 = Bothnian Bay, 7 = Bothnian Sea, 8 = Åland Islands, freshwater and coastal area, 9 = Archipelago Sea (eastern parts), 10 = Gulf of Finland. SE = Sweden, FI = Finland, RU = Russia

Fish samples from farms located in both the inland freshwater areas of Finland and in the Finnish coastal area of the Baltic Sea were sent to the Finnish Food Safety Authority (Evira) for viral investigation (Fig. 1). The farms included 55 sea cage farms producing food fish or ongrowing juveniles for food fish purposes, or a combination of these, and 17 freshwater farms, including a hatchery, broodstock fish farms, and farms producing both juveniles and food fish. Most farms were a combination of different types of production systems. In addition, two freshwater recirculation aquaculture system (RAS) farms producing food fish or broodstock fish and juveniles were included. In two cases, the viral isolates originated from wild fish caught for broodstock renewing purposes. The fish were sampled for miscellaneous health screening purposes or the investigation of clinical disease. The fish samples studied consisted of whole fish on ice, live fish, pooled organ samples or ovarian fluids. The whole fish were necropsied, and pooled organ samples consisting of (head or mid-) kidney, spleen and (heart or) brain tissue were used for virus isolation by cell culture. Most of the fish tested were rainbow trout (*Oncorhynchus mykiss*). However, a few samples were taken from other fish species, namely brown trout (*Salmo trutta*), Atlantic salmon (*Salmo salar*) and whitefish (*Coregonus lavaretus*). Viral VP2 gene sequence data for isolates from the period 2012–2014 that were published in our previous work [21] were included in the analyses described here.

### Virus isolation in cell culture

Virus isolations were carried out from pooled organ samples or ovarian fluids. Pooled heart or brain, anterior kidney and spleen tissue from a maximum of 10 fish were homogenised in 9 volumes of cell culture medium (Eagle’s MEM, Gibco plus 8 to 10% foetal bovine serum; pH 7.2 to 7.4) containing penicillin and streptomycin. Homogenates were centrifuged (15 min, 4000 × *g*, 4 °C) and the supernatants inoculated onto subconfluent monolayer cell cultures of bluegill fry (BF-2; [22]) and epithelioma papulosum cyprini (EPC; [23]) cultures in 24-well plates (Nunc A/S) at final dilutions of 1:100 and 1:1000. Before 2009, the samples were incubated at 16 °C in CO_2_. After 2009, the samples were buffered with sodium carbonate 7.5% (Gibco 25080) and incubated without CO_2_. The susceptibility of the cell lines to different viruses was tested biannually during the study period by standardised titration procedure. The samples were inspected regularly under a microscope for the occurrence of cytopathic effect (CPE). After 7 d of incubation, supernatant from samples without CPE was diluted 1:100 and 1:1000, sub-cultured onto fresh cells and incubated for a further 7 d. When CPE was observed, the supernatant was collected and stored at −70 °C for future studies.

### Detection of viruses with ELISA

Aliquots of 50 µl of culture medium from cell cultures showing evidence of CPE were analysed with commercial ELISA kits according to the manufacturer’s instructions to test for the presence of IPNV, spring viraemia of carp virus (SVCV), viral haemorrhagic septicaemia virus (VHSV) (Test-Line Ltd., Brno, Czech Republic), and since 2008, also for the presence of infectious hematopoietic necrosis virus (IHNV) (Bio-X Diagnostics S.P.R.L, Jemelle, Belgium).

### RNA extraction

For PCR and subsequent Sanger sequencing, the infected cell culture monolayers were harvested and total RNA was extracted using the QIAamp Viral RNA Mini Kit (Qiagen, Hilden, Germany) according to the manufacturer’s instructions. For next generation sequencing, the total RNA was extracted using the RNeasy Mini Kit (Qiagen). During RNA extraction, the RNase-Free DNase Set (Qiagen) was used to remove DNA from the samples.

### PCR

A real-time PCR method with forward primer VP3F: 5’-CGACCGACATGAACAAAATCA-3’, reverse primer VP3R: 5’-TGTGCGAATACAGCTGCAACT-3’, and VP3 probe: 5’- FAM-TCTAGCCAACAGTGTGTACGGCCTCCC-BHQ1-3’ [24]) was used to amplify and detect a 109-base-pair (bp) fragment of the IPNV VP3 gene. The nucleotide (nt) positions of the primers and probe according to a previously published IPNV isolate Sp122 segment A (NCBI GenBank accession number AY354521) were the following: VP3F 5’ (2754) – 3’ (2774); VP3R 5’ (2862) – 3’ (2842); VP3 probe 5’(2779) – 3’(2805). Primers F (5’-ACGAACCCCCAGGACAA-3’, modified from McColl et al. [25]) and A2 (5’- GACAGGATCATCTTGGCATAGT-3’, [26]) were used to amplify 776 bp of the IPNV VP2 gene. The nt positions for the VP2 gene primers according to the IPNV isolate Sp122 segment A were as follows: F 5’(554) – 3’(570) and A2 5’(1329) – 3’(1308). For both PCR assays, a one-step reverse transcription (RT)-PCR reaction was performed using the Qiagen OneStep RT-PCR Kit according to the manufacturer’s instructions, with 3 µl (VP3 real time RT-PCR) and 5 µl (VP2 RT-PCR) of RNA as a reaction template.

### Sequencing and data analysis

PCR products were purified from agarose gels using the MinElute Gel Extraction Kit (Qiagen). Both strands of the PCR products were sequenced using the Big Dye Terminator v1.1 Cycle Sequencing Kit (Applied Biosystems, Foster City, CA, USA) and an Applied Biosystems 3130 Genetic Analyzer instrument.

For next generation sequencing (NGS), RNA samples were sent to the Institute for Molecular Medicine Finland (FIMM) in Helsinki, where libraries were prepared and sequenced using an Illumina MiSeq instrument [27], and the raw paired-end sequence data (2 x 150 bp) were trimmed, followed by *de novo* analysis for genome assembly using the ABySS assembler [28]. The results of the *de novo* analyses were further analysed using BLAST to identify the contig sequences containing aquabirnavirus genome sequences. The sequences for genome segments A and B were constructed by compiling the contig sequences using GeneDoc v2.7. Additionally, the trimmed NGS data were assembled by read mapping to reference sequences (GenBank accession numbers for A segment: AY780919, AY354521; B segment: AY780926, AJ622823) using CLC Genomics Workbench 8.5.1. (Qiagen). The sequences for segments A and B obtained from the *de novo* analyses and from the read mapping to reference sequences were compared and any gaps detected in the sequences were filled by designing specific primers and using Sanger sequencing.

Phylogenetic analyses for the partial VP2 gene sequences and for the complete coding sequences of A and B segments were performed with MEGA 7.0.7 [29] using the maximum likelihood method based on the general time-reversible model. The reliability of the analyses was assessed by bootstrap with 1000 replicates. In addition to the other previously published IPNV sequences, the VP2 gene sequences of 85 IPNV isolates from the period 2012–2014 published in a previous study [21] were included in the analyses in order to describe the genetic properties of the Finnish IPNV populations during the whole period from 2000 to 2015. The GenBank accession numbers for the previously published sequences used were: AF342735 (Jasper), AF343572 (VR299), AF342729 (Ab), AY780919 (6B1A), AF342732 (Canada 1), AF342731 (Tellina virus 2), AF342733 (Canada 2), AF342734 (Canada 3), AJ622822 (Sp 31-75), AJ489229 (88R), AF342730 (Hecht), AY283781 (Y-6) and AY283783 (H1), and for the Finnish IPNV isolates: KR780984 (ka89/12), KR780985 (ka893/12), KR780986 (ka890/12), KR780987 (ka124/12), KR780988 (ka745/12), KR780989 (ka391/13), KR780991 (ka530/13), KR780993 (ka103/14), KR780994 (ka251/14), KR780995 (ka459/14), KR780996 (ka940/14), KR780997 (ka1005/14), KR780998 (ka1049/11), KR780999 (ka568/12), KR781000 (ka844/12), KR781001 (ka692/13), KR781002 (ka735/13), KR781003 (ka764/14), KR781004 (ka954/14), KR781005 (ka978/14), KR781006 (ka1038/14), KR781007 (ka639/12), KR781008 (ka640/12), KR781009 (ka666/12), KR781010 (ka1496/12), KR781011 (ka1613/12) and KR781012 (ka798/13).

The pairwise nucleotide distance of the partial VP gene between all studied isolates and the first inland isolate ka89/12 in 2012 was calculated as the number of nucleotide differences with MEGA. The Kruskal Wallis test was used in multiple group comparisons of nucleotide differences comparing the geographical origin of the isolates, farm type and line of production at the farm. Dunn’s test was used for *post hoc* pairwise multiple comparisons. Statistical analyses were performed with the GraphPad Prism 6 software. Percent similarity values for sequence pairs were calculated using the MegAlign program from the DNASTAR Lasergene 10 application package. Tajima’s D test of neutral evolution was conducted with DnaSp 5.10.01 software to detect natural selection in the partial VP2 gene nucleotide sequences. Each dataset tested contained sequences of viruses isolated from the same geographic region in a single year. Because Tajima’s D requires at least four sequences for its calculation, data from two consecutive years were combined into one dataset in cases where less than four isolations had been made in a year.

## Results

### Virus isolation and identification with ELISA

IPNV was isolated from 88 pooled samples from rainbow trout, brown trout, whitefish and Atlantic salmon from 10 different geographical areas during 2000–2011 and 2014–2015 (Table 1). In addition, an isolate from 1989 was included in the study, as there was previous knowledge that the isolate belonged to genogroup 6 (Hecht, serotype A4). Altogether, a total of 173 isolates were used in the genetic analyses, including sequences from a previous study [21]. Of these isolates, 155 originated from rainbow trout, 11 from brown trout, 4 from Atlantic salmon, and 3 from whitefish. The annual number of IPNV isolations was relatively low before 2010, with the exception of 2007, when IPNV was isolated from 8 coastal farms. After the introduction of genogroup 2 IPNV into inland farms in 2012, the number of isolations increased and reached the highest level in 2015, with 22 affected farms. Isolates were initially identified by the cytopathic effect, typical of IPNV, which they induced on BF-2 and EPC cells. All isolates studied grew in cell culture, and of these, 71 isolates were positive in the IPNV ELISA test. After conventional PCR and Sanger sequencing, the 11 isolates that were negative in IPNV ELISA were found to be IPNV genogroup 6 isolates. All samples studied were negative for IHNV, SVCV and VHSV in ELISA tests.

**Table 1:**
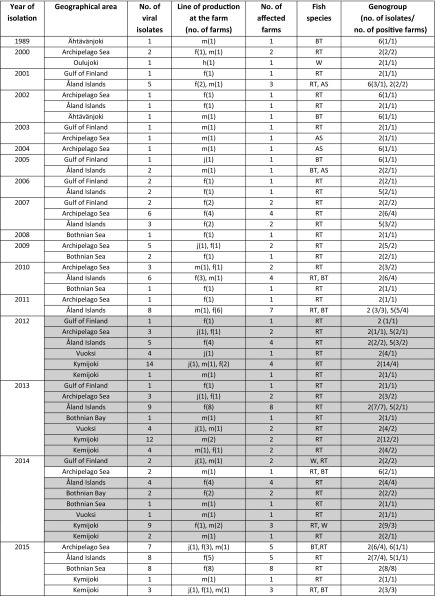
Finnish IPNV isolates from 1989 and 2000–2015

f = food fish farm, m = mixed production producing broodfish, juveniles and/or food fish, j = juvenile production, h = hatchery. RT = rainbow trout *Oncorhynchus mykiss*, BT = brown trout *Salmo trutta*, W = whitefish *Coregonus lavaretus*, AS = Atlantic salmon *Salmo salar*. Isolates published by Eriksson-Kallio et al. [21] are indicated with grey shading

### PCR and Sanger sequencing of the partial VP2 gene

**Fig. 2:**
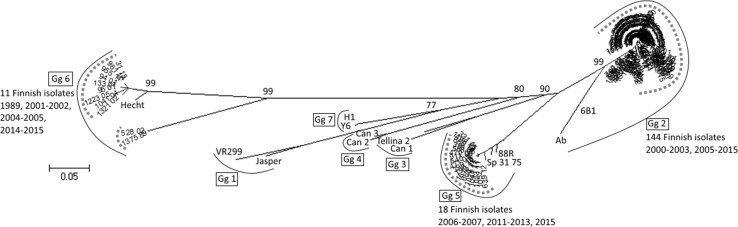
Maximum likelihood analysis based on partial IPNV VP2 gene sequences. Finnish IPNV isolates are marked with a dotted curved line. Genogroups (Gg) are marked with a solid curved line. The number of isolates and the year of isolation of the Finnish isolates are given under each genogroup. The scale bar indicates the number of substitutions per site. Numbers at the nodes of the tree indicate bootstrap values; values higher than 70 are given. The GenBank accession numbers of previously published sequences used in the analysis are presented in the “Materials and methods”

The partial VP2 gene was successfully amplified from all 88 isolates using the conventional VP2 RT-PCR with primers F and A2. The primer A2 was originally published by Bain et al. [26], and was used to amplify an 1180 bp fragment of the VP2 gene, whereas in this study, a fragment of 767 bp was amplified, sequenced and analysed. Despite the shorter sequences used here, the clustering of isolates of different genogroups remained similar in the phylogenetic analysis, when compared to the results reported by Bain et al. VP2 sequences of a total of 173 Finnish IPNV isolates were used in the genetic analyses, including sequences from a previous study [21]. Based on the phylogenetic analysis (Fig. 2), the Finnish isolates could be categorized into three genogroups; 144 isolates belonged to genogroup 2, 18 isolates to genogroup 5 and 11 isolates to genogroup 6, according to Blake et al. [18] and Nishizawa et al. [19]. The grouping published by Blake et al. was based on a 1611 bp fragment of the VP2 gene, and grouping by Nishizawa et al. was based on a 358 bp fragment of the VP2/NS region. Between these two publications there were differences in the grouping of isolates into genogroups 3, 4 and 5. According to Blake et al., isolate Tellina belonged to genogroup 3, isolates Canada 2 and 3 into genogroup 4, and isolate Sp into genogroup 5, whereas according to Nishizawa et al., isolate Tellina belonged to genogroup 4, isolates Canada 2 and 3 into genogroup 5, and isolate Sp into genogroup 3. In this study, genogroups 3, 4 and 5 were defined as in Blake et al. Altogether 11 isolates were negative in the VP3 real time RT-PCR. All these isolates belonged to genogroup 6 based on the partial VP2 sequences. Based on the polyprotein sequences obtained from the two Finnish genogroup 6 isolates 1379/89 and 94/01, there were 6 nucleotide differences at the annealing site of the primer VP3F in the viral genome compared to the primer sequence. This most likely explains why the VP3 real time RT-PCR was not able to amplify the genogroup 6 isolates.

In the inland area, only genogroup 2 isolates were found, with the exception of two isolations of highly similar genogroup 6 viruses in 1989 and 2002 from the Ähtävänjoki river system. In Ähtävänjoki, the sampling site was situated close to the Bothnian Bay, but several physical barriers hinder the migration of fish. In general, the Finnish isolates appeared to form their own subgroup within genogroup 2. Genogroup 5 isolates formed a more uniform cluster with previously published isolates Sp 31-75 (GenBank accession number AJ622822) and 88R (GenBank accession number AJ489229), whereas in genogroup 6, two subgroups were revealed. The subgroups consisted of Finnish isolates genetically closely related to the isolate Hecht and more divergent isolates from Ähtävänjoki. Pairwise VP2 gene nucleotide sequence identity values between the Hecht-like and the Ähtävänjoki isolates varied between 80.5 – 81.4%. In the area of the Åland Islands and the Archipelago Sea, isolates of all three genogroups were present, whereas in the coastal regions of the Gulf of Finland, genogroup 2 and 6 isolates were found. In the Bothnian Sea and Bothnian Bay regions, only genogroup 2 viruses were isolated. In general, genogroup 2 isolates were the most abundant and they were detected annually throughout the study period, whereas identification of genogroup 5 and 6 isolates was more irregular. Identical genogroup 2 VP2 sequences were detected at the same farm on three different occasions: in two consecutive years both in the Gulf of Finland (2006 and 2007) and Archipelago Sea (2009 and 2010), and in 2013 and 2015 in the Åland Islands. In 2015, an identical isolate was detected in three coastal areas (Åland Islands, Archipelago Sea and Bothnian Bay) at seven separate farms. This isolate type was first detected in the Åland Islands in 2011, after which it was found at several freshwater and coastal farms in 2012–2014 [21].

Recurrent viral infections that occurred during different years on the same farm were detected at several coastal and inland farms. Recurrence was observed with all three IPNV genogroups. Additionally, at some farms, isolates of two different genogroups, either genogroup 2 and 5 or 2 and 6, were detected during the study period.

Genogroup 2 isolates were found in all fish species included in this study, whereas genogroup 5 isolates were only found in rainbow trout and genogroup 6 isolates in rainbow trout, brown trout and Atlantic salmon. Genogroup 2 isolates identical in the VP2 sequence were found in the same year (2000) in whitefish and rainbow trout from two geographically distant locations: Oulujoki and the Archipelago Sea, respectively. The Oulujoki whitefish samples originated from wild brood fish. Similarly, in 2003, genogroup 2 isolates with identical VP2 sequences were found in Atlantic salmon and rainbow trout in the Archipelago Sea and the Gulf of Finland, respectively.

Pairwise VP2 gene nucleotide differences between all isolates from the years 1989 and 2000–2015, and the first inland isolate (ka89/12) found in 2012, were calculated as the number of differing nucleotides between two sequences and used in group-wise comparisons of viruses from different geographical origins, and from different farm types and lines of production. Descriptive information on the new and previously published Finnish IPNV isolates is presented in Table 1. The difference in the nucleotide composition of isolates from the Åland Islands and Archipelago Sea compared to the isolates from Kymijoki and Vuoksi was statistically significant (p < 0.001). Moreover, isolates from Vuoksi differed from isolates from Ähtävänjoki and from the Gulf of Finland (p = 0.01 and 0.03, respectively). When comparing the farm types, the isolates from sea cages differed from freshwater farm isolates (p < 0.001). Additionally, a difference was observed between isolates from farms producing juveniles and isolates from both food fish farms and farms with mixed production (p = 0.007 and 0.03, respectively).

Tajima’s D values indicating balancing selection or population subdivision were obtained from VP2 gene sequences of Åland Island isolates collected in 2010–2011 (D = 2.06, p < 0.05) and in 2012 (D = 1.87, p < 0.05). Values indicating purifying selection or rapid population growth were obtained from isolates collected in 2015 from the Åland Islands (D = -1.86, p < 0.01) and the Archipelago Sea (D = -1.75, p < 0.01).

Amino acid patterns in the polyprotein suggested to be involved in pathogenicity [7, 14, 15] were also examined. The most common pattern found in genogroup 2 and 5 isolates was proline at position 217 (Pro217), threonine at position 221 (Thr221) and alanine at position 247 (Ala247). Additionally, patterns Ser217-Thr221-Ala247 and Pro217-Thr221-Gln247 with no reference to pathogenicity in the literature were detected. The latter pattern was only found in genogroup 6 isolates, whereas the former was found in a total of 19 genogroup 2 isolates.

### Sequencing of the complete coding regions of viral genome segments A and B

Based on the phylogenetic analysis of the partial VP2 gene sequences, a total of 11 isolates representing genogroups 2, 5 and 6 were chosen for next generation sequencing (Table 2). The coding sequences of the genome segments A and B were obtained from all 11 isolates.

**Table 2:** IPNV isolates that were analysed at the complete polyprotein and VP1 coding region level

Isolate ID	Origin of the isolate	Year of isolation	Fish species	Passage in cell culture	Genogroup	G+C content (%)	GenBank accession numbers	
							Segment A	Segment B
1375/89	Ähtävänjoki	1989	BT	6	6	52.3	KY548508	KY548519
94/01	Åland Islands	2001	AS	4	6	52.6	KY548509	KY548520
284/01	Åland Islands	2001	RT	5	2	54.7	KY548510	KY548521
470/07	Åland Islands	2007	RT	2	5	54.7	KY548511	KY548522
247/10	Archipelago Sea	2010	RT	4	2	54.8	KY548512	KY548523
90/12	Kymijoki	2012	RT	2	2	55.0	KY548513	KY548524
639/12	Archipelago Sea	2012	RT	2	5	54.9	KY548514	KY548525
666/12	Åland Islands	2012	RT	2	5	54.7	KY548515	KY548526
745/12	Kymijoki	2012	RT	2	2	54.9	KY548516	KY548527
844/12	Archipelago Sea	2012	RT	3	2	54.7	KY548517	KY548528
890/12	Kemijoki	2012	RT	3	2	54.9	KY548518	KY548529

BT = Brown trout *Salmo trutta*, AS = Atlantic salmon *Salmo salar*, RT = Rainbow trout *Oncorhynchus mykiss*

**Fig. 3:**
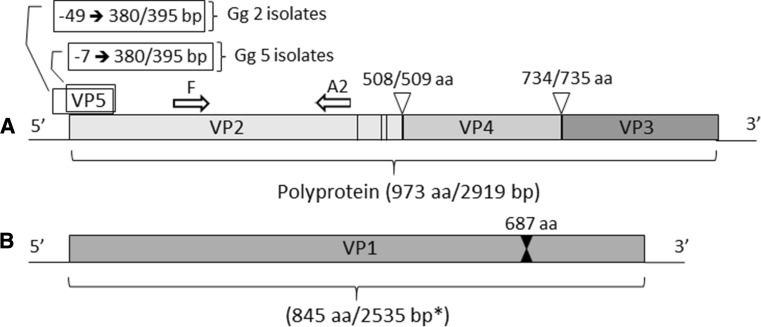
A and B segments of the Finnish IPNV isolates. The amino acid (aa) positions for the cleavage sites of the polyprotein are marked with arrowheads and vertical lines. The position and length of the VP5 ORF varies between isolates and genogroups (Gg), and is given in nucleotides in relation to the polyprotein ORF. *The length of the VP1 ORF was 845 aa in all isolates except for 247/10, in which the aa in position 687 was absent. The annealing positions of the primers F and A2 used to amplify a 767 bp fragment of the VP2 are indicated with arrows

The ORF for the polyprotein in segment A was 2919 bp (973 amino acids) in length in all the studied isolates (Fig. 3). The cleavage sites for the IPNV VP4 protease N and C termini were found after amino acids 508 and 734, respectively, as reported previously [30, 31]. Similarly, the cleavage sites within pVP2 were located after amino acid residues 442, 486 and 495. The motifs within pVP2 and at the pVP2-VP4 and VP4-VP3 junction were Ser/Thr – X – Ala ↓ Ser/Ala – Gly, as described by Petit et al. [32]. The length and positioning of the ORF encoding the VP5 protein in segment A varied between isolates. In genogroup 2 isolates, the length of VP5 ORF was 444 bp (isolates 284/01, 90/12, 745/12, 890/12) or 429 bp (isolates 247/10, 844/12), whereas in genogroup 5 isolates, the length was 387 bp (isolate 639/12) or 402 bp (isolates 470/07 and 666/12). In all isolates, the VP5 ORF overlapped the polyprotein, but the length of the ORF and the location of the start codon varied between genogroups. The VP5 ORF was not found in the genogroup 6 isolates 1375/89 and 94/01.

**Fig. 4:**
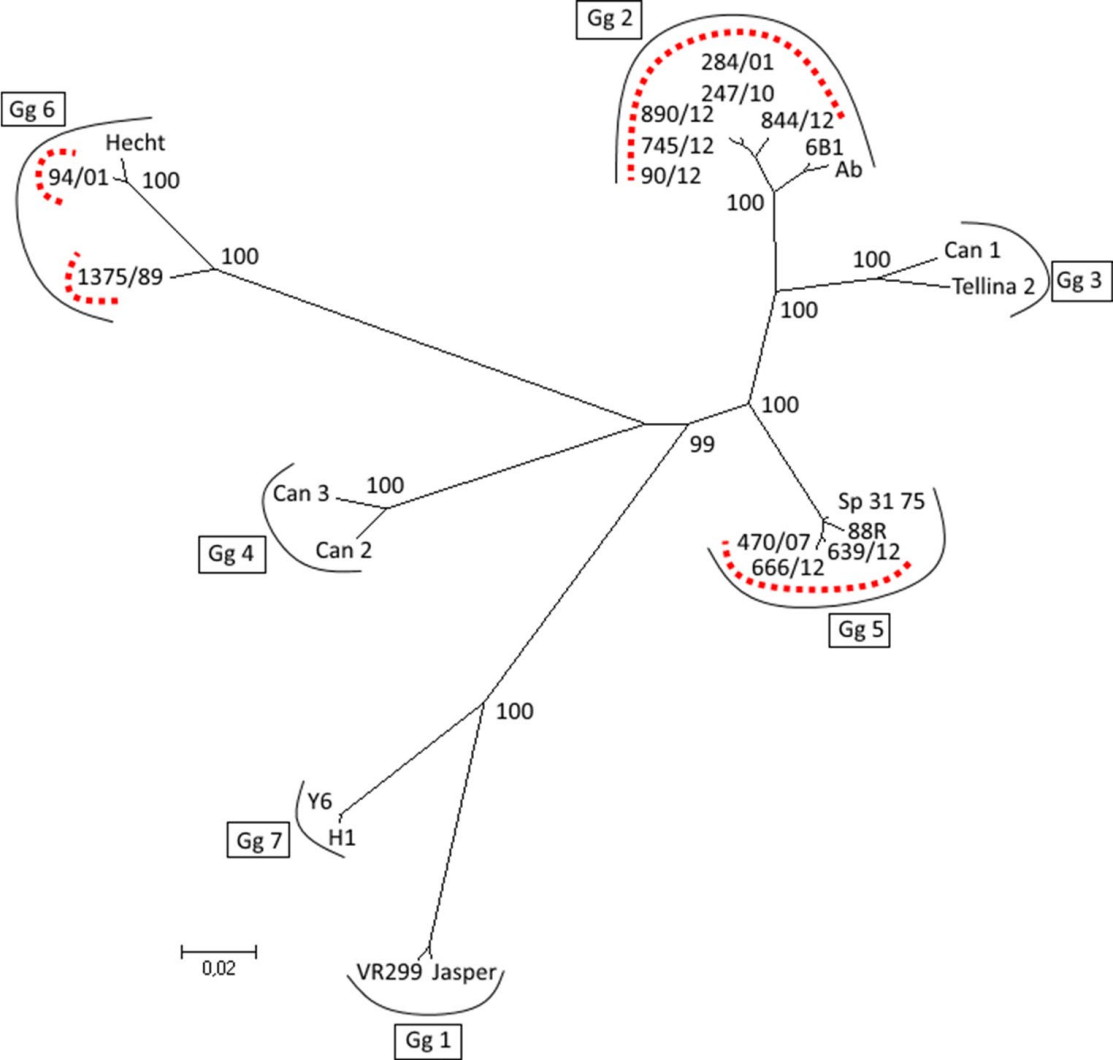
Maximum likelihood analysis based on IPNV polyprotein sequences. The Finnish isolates are marked with a dotted curved line, and genogroups (Gg) with a solid curved line. The scale bar indicates the number of substitutions per site. Numbers at the nodes of the tree indicate bootstrap values; values higher than 70 are given. The GenBank accession numbers of previously published sequences used in the analysis are presented in the “Materials and methods”

Phylogenetic analysis based on the polyprotein sequences (Fig. 4) revealed similar grouping of isolates as the analysis based on the partial VP2 gene. Likewise, phylogenetic analysis based on VP1 sequences (data not shown), as well as the pairwise sequence identity values of both polyprotein and VP1 sequences (Table 3), confirmed the clustering of isolates. Based on the polyprotein and VP1 sequences, no viral reassortment was detected among the isolates. The overall sequence pair similarity among Finnish genogroup 2 isolates varied between 98.3% and 99.9% at the nucleotide level and 98.7% and 100% at the amino acid level for the polyprotein and VP1 gene, respectively. Among genogroup 5 isolates, the similarity values for nucleotide sequences were 98.8% to 99.7% and for the amino acid sequences 99.5% to 100%. Between the two genogroup 6 isolates 1375/89 and 94/01, higher genetic diversity was detected, with nucleotide sequence similarity values of 84.9% and 85.4% for the polyprotein and VP1, respectively. These results supported the subgrouping of the genogroup 6 isolates that was detected based on VP2 sequences.

**Table 3:**
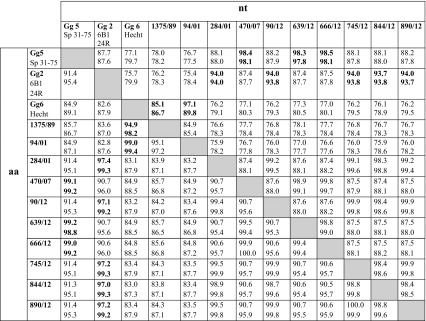
Sequence pair similarity values (percentages, %) based on the nucleotide (nt) and amino acid (aa) sequences of the polyprotein and VP1 coding regions of the IPNV isolates

Nucleotide sequence identity values are presented in the upper diagonal, and amino acid sequence identity values in the lower diagonal part of the table. In each cell, the upper value is for the polyprotein and the lower value is for the VP1 sequences. NCBI Genbank Accession numbers for previously published polyprotein sequences: Sp 31-75 (AJ622822), 6B1 (AY780919), Hecht (AF342730), and for the VP1 sequences: Sp 31-75 (AJ622823), 24R (AJ489243), Hecht (JF734351, partial VP1 sequence: 498bp). The highest similarity values for each Finnish IPNV isolate are in bold. Gg = genogroup

The VP1 ORF in segment B was 2535 bp in all isolates except for isolate 247/10, in which the coding region was 2532 bp. Only a partial VP1 sequence of genogroup 6 IPNV (isolate Hecht) was available in the NCBI GenBank for genetic analysis and sequence comparisons. Based on both polyprotein and VP1 sequences, isolate 94/01 showed high similarity with isolate Hecht, whereas isolate 1375/89 appeared to be more divergent at both the nucleotide and amino acid sequence level.

The overall G+C content of the coding genomic regions of the isolates studied varied between 52.3% and 55%; the genogroup 6 isolates 1375/89 and 94/01 exhibited a lower G+C content than the genogroup 2 and 5 isolates.

## Discussion

As a continuation of our previous research focusing on the period from 2012–2014 [21], this study describes the molecular analysis of IPNV isolates collected from Finnish fish farms during 2000–2011 and 2015. Based on the genetic analyses, IPNV genogroups 2, 5 and 6 were present at the fish farms during the study period. In general, the genetic diversity within the Finnish genogroups 2 and 5 was relatively low, whereas within genogroup 6, two subgroups were revealed. Based on the polyprotein and VP1 sequences, the genetic distance between genogroup 6 isolates and genogroup 2 and 5 isolates appeared to be relatively high. This was most likely the reason why the genogroup 6 isolates were not detected with ELISA or with the VP3 real time RT-PCR used in this study as these methods were developed mainly for the detection of genogroup 5 IPNV. Genogroup 2 isolates were the most abundant at both the inland and coastal farms. Genogroup 6 occurred sporadically, and was generally isolated from farms taking in fish material originating from wild fish.

The main cultured food fish species in Finland [33], and therefore the most common species sent for viral sampling to Evira, is rainbow trout. Thus, 154 of the 173 IPNV isolates collected during 2000–2015 originated from rainbow trout. Genogroup 2 and 6 IPNV isolates were found in several salmonid fish species, whereas genogroup 5 isolates were only found in rainbow trout. In addition to genogroup 2, only two isolations of genogroup 6 viruses in the Ähtävänjoki river system close to the coast of Bothnian Bay were made from the inland farms. At the coastal farms of the Åland Islands and Archipelago Sea, isolates of all three genogroups were found.

Besides the geographic origin, the farm type and line of production appear to have a relationship with the genetic properties of the isolates present at the farm. In general, Finnish fish production is divided into two geographical areas: the production of broodstock and juveniles in freshwater inland farms, and the production of food fish at brackish water coastal farms, with some minor exceptions. In Finland, the majority of rainbow trout originate from domestic broodfish. From the end of 1980s to 1998, import of live fish and eggs to Finland was forbidden, and since 1998, only fish and eggs from IPN-free sources have been allowed to be imported. The IPNV isolates found at the broodstock and juvenile farms seemed to differ genetically from the food fish farm isolates. As described previously [21], the source of genogroup 2 IPNV introduced to inland farms in 2012 was most likely one or several coastal farms. The genogroup 2 isolate type that was first detected in the Åland Islands in 2011 was later found at several inland and coastal farms in 2012–2015. The annual number of IPNV isolations in the coastal farms increased after the introduction of genogroup 2 IPNV to the inland farms. This can most likely be explained by the transfer of juvenile fish from inland farms to food fish farms in the coastal area. The results obtained from Tajima’s D test in this study support the theory of an increased viral population size in the coastal area of the Åland Islands and Archipelago Sea in 2015. Most of the viral isolates in this study were obtained from the Åland Islands and Archipelago Sea, reflecting the fact that the majority of food fish farms are located in this area. In addition, the mixing of fish from different sources is common, enabling the spread of viral isolates between farms. However, in order to prevent the spread of genogroup 5 isolates to inland areas, the transfer of fish from coastal areas to inland is restricted by legislation.

In this study, complete genomic coding sequence was obtained from 11 isolates from all three IPNV genogroups observed. Prior to this study, only partial VP1 sequences of genogroup 6 IPNV were available in the NCBI GenBank. The phylogenetic clustering based on complete coding sequences of genomic segments A and B of Finnish IPNV isolates was in accordance with the results obtained from VP2 gene sequences. This indicates that no genetic reassortants were among the isolates studied although fish from different sources are often mixed. Reassortment is an important mechanism of evolution in dsRNA viruses [34], and natural reassortment has been shown to occur in IPNV [35]. The isolates studied here showed variance in the length and positioning of the VP5 ORF in genomic segment A. In general, the genogroup 5 isolates exhibited shorter VP5 ORFs than the genogroup 2 isolates, whereas in the genogroup 6 isolates, no VP5 ORF was found. The absence of VP5 in genogroup 6 isolate Hecht has previously been reported [36]. Additionally, it has been shown that VP5 is not needed for viral replication, and that its absence does not affect the virulence of the virus or the establishment of persistent IPNV infection [37, 38].

The association of certain amino acid patterns in the viral polyprotein and virulence of genogroup 5 (serotype Sp) IPNV isolates has been described in several publications [14, 15, 39]. As shown previously with the Finnish IPNV isolates from 2012–2014 [21], most of the genogroup 2 and 5 isolates in this study demonstrated the amino acid pattern Pro217-Thr221-Ala247. In Scotland and Ireland, the same amino acid pattern has been detected in genogroup 5 isolates in connection with clinical outbreaks [26, 40], whereas in Norway, the pattern has been associated with avirulence [41] or subclinical disease [39]. All genogroup 6 isolates in this study exhibited amino acids Pro217-Thr221-Gln247, a pattern that has neither been connected with virulence nor avirulence in the literature. No severe IPN outbreaks have so far occurred in Finland. However, increased mortality rates in coinfections with IPNV and bacterial diseases have been noted frequently [21]. In addition, mortalities of susceptible fry showing histopathological findings consistent with IPN with no association with other infectious agents than IPNV have been reported. Apart from reports of genogroup 1 IPNV from Chile [42, 43], where the isolates from clinical outbreaks had the amino acid pattern Ala217-Thr221, little information is available on the virulence-related amino-acid motifs of IPNV genogroups other than genogroup 5. Virulence and mortality depends not only on the genetic properties of the virus, but also on factors related to the host immune system [44]. Fish are believed to develop resistance to IPNV infection with age [45]. Ortega et al. [43] reported that the genotype and the infectivity of different IPNV isolates does not translate into clear differences in the antiviral responses in the host. Additionally, it has been shown that in persistently infected salmon, environmental stress can lead to changes where attenuated, nonvirulent IPNV variants become more virulent [46]. It is evident that experimental infection trials are needed in order to obtain more information on the genetic properties related to the virulence of isolates of different IPNV genogroups.

Fish surviving IPNV infection become asymptomatic persistent carriers of the virus [47], and carrier fish are very common [48]. Both virulent and avirulent IPNV isolates are able to establish persistent infection [49]. In carrier fish, IPNV has been reported to reside and multiply within head kidney-derived leukocytes [50, 51]. It has been suggested that IPNV infects B lymphocytes and causes immunosuppression, resulting in a carrier state [52], and that the viruses attenuate the antiviral responses in the host at different levels which may cause differences in the disease outcome [49]. Additionally, it has been shown that in the carrier state, the virus titre can vary with time from non-detectable to comparatively high [53–55]. Here, the two highly similar genogroup 6 isolates found in brown trout in Ähtävänjoki in 1989 and 2002 indicate that the virus is able to remain on the same farm for years. Wild fish carriers of IPNV cannot be ruled out as a source of infection because of the lack of systematic, targeted surveys on the occurrence of IPNV in wild fish in Finnish waters. However, wild salmonid broodfish and other wild salmonids sent to Evira, both from the sea area and the inland area, are tested annually for IPNV, and no IPNV has been found in inland waters [21]. It has been show that IPNV can be isolated from mussels, sediment and surface water in the vicinity of clinically infected farms [56]. However, there is little information on whether IPNV released from environmental reservoirs could re-infect or sustain infection on fish farms. As shown in this study, IPNV appears to be widely spread among Finnish fish farms, and the number of annual viral isolations has recently increased. It has been shown that the prevalence of IPNV can differ between years [57]. On the other hand, the prevalence at marine sites may be underestimated and the virus could be ubiquitous [58]. In this study, the recurrence of viral infection at the same farm was detected in several coastal and inland farms, indicating either persistent infection or reinfection. In the inland broodstock and juvenile production farms, the infection is most likely sustained in fish that can be held at the same location for longer periods of time. In the coastal food fish farms, the fish material is regularly replaced, and recurring infections most likely originate from incoming fish stock or from the environment.

In conclusion, IPNV appears to be a highly prevalent pathogen on Finnish fish farms, and genogroup 2 is geographically more widely spread than genogroups 5 or 6. In coastal food fish farms, all three genogroups were found, whereas in the inland broodstock and juvenile producing farms, only genogroup 2 was regularly detected. Genogroups 2 and 5 appear to be genetically more uniform than genogroup 6. All isolates studied here demonstrated amino acid patterns previously associated with avirulence in genogroup 5. However, more research is needed to clarify the relationship between pathogenicity and genetic properties of different IPNV genogroups.
